# Skeleton Reconstruction Using Generative Adversarial Networks for Human Activity Recognition Under Occlusion

**DOI:** 10.3390/s25051567

**Published:** 2025-03-04

**Authors:** Ioannis Vernikos, Evaggelos Spyrou

**Affiliations:** Department of Informatics and Telecommunications, University of Thessaly, 35100 Lamia, Greece; ivernikos@uth.gr

**Keywords:** human activity recognition, occlusion, reconstruction of skeleton joints, generative adversarial networks, convolutional neural networks

## Abstract

Recognizing human activities from motion data is a complex task in computer vision, involving the recognition of human behaviors from sequences of 3D motion data. These activities encompass successive body part movements, interactions with objects, or group dynamics. Camera-based recognition methods are cost-effective and perform well under controlled conditions but face challenges in real-world scenarios due to factors such as viewpoint changes, illumination variations, and occlusion. The latter is the most significant challenge in real-world recognition; partial occlusion impacts recognition accuracy to varying degrees depending on the activity and the occluded body parts while complete occlusion can render activity recognition impossible. In this paper, we propose a novel approach for human activity recognition in the presence of partial occlusion, which may be applied in cases wherein up to two body parts are occluded. The proposed approach works under the assumptions that (a) human motion is modeled using a set of 3D skeletal joints, and (b) the same body parts remain occluded throughout the whole activity. Contrary to previous research, in this work, we address this problem using a Generative Adversarial Network (GAN). Specifically, we train a Convolutional Recurrent Neural Network (CRNN), whose goal is to serve as the generator of the GAN. Its aim is to complete the missing parts of the skeleton due to occlusion. Specifically, the input to this CRNN consists of raw 3D skeleton joint positions, upon the removal of joints corresponding to occluded parts. The output of the CRNN is a reconstructed skeleton. For the discriminator of the GAN, we use a simple long short-term memory (LSTM) network. We evaluate the proposed approach using publicly available datasets in a series of occlusion scenarios. We demonstrate that in all scenarios, the occlusion of certain body parts causes a significant decline in performance, although in some cases, the reconstruction process leads to almost perfect recognition. Nonetheless, in almost every circumstance, the herein proposed approach exhibits superior performance compared to previous works, which varies between 2.2% and 37.5%, depending on the dataset used and the occlusion case.

## 1. Introduction

Without a doubt, the task of identifying human activities from motion data stands as one of the most challenging tasks in the broad research field of computer vision. This task can be described as the process of recognizing human behavior within a sequence of images or videos, using visual data, obtained from the motion of the human subjects in the 3D space. These behaviors include a series of successive body part movements, commonly known as “actions” or activities. Specifically, an action may be defined as a unique type of motion carried out by a human, usually of short duration [1]. Actions are not instantaneous and often involve several body parts. This category also comprises interactions, either between humans and objects or among multiple individuals involved in group activities. It should be noted that in the context of this paper, (a) the term “activity” would be used to collectively refer to all the aforementioned categories; (b) gestures, which are short and typically involve only a few body parts will not be considered; and (c) experiments will be based on segmented sequences, each containing exactly a single action to be recognized.

Camera-based Human Activity Recognition (HAR) methods are generally cost-effective since they could rely on low-cost off-the-shelf hardware, while they exhibit remarkable performance under laboratory settings. However, their performance tends to degrade in real-world scenarios due to three primary factors, i.e., changes in viewpoint/illumination, and occlusion. Variations in viewpoint may occur, for instance, when the subject is observed from different angles than the one(s) used during training. To address this, in previous work [2], it has been experimentally proven that the accuracy drop due to viewpoint changes may be mitigated by employing multiple cameras. Moreover, changes in illumination, and especially those leading to low-light conditions, predominantly affect video-based methods. Nevertheless, recent technological advances have led to the development of camera sensors capable of capturing depth information (which is unaffected by changes in illumination), thereby significantly improving performance under low-light conditions. In such cases, combining video and depth data can facilitate robust extraction of human silhouettes, for instance, as a 3D point set [3].

Therefore, occlusion remains the most significant challenge among the three issues discussed previously. In real-world conditions, it is typically caused by furniture [4], other people present [5], or even by the same subject (i.e., self-occlusion) [6], while complete occlusion of some body parts renders activity recognition impossible. Partial occlusion, however, can still affect the accuracy of recognition to some point, which varies, depending on the activity and the body part(s) affected [7]. In previous work [8], this problem has been addressed by treating it as a regression task solved using a deep neural network. This network was trained so as to reconstruct the missing skeleton joints, therefore providing a “complete” skeleton. Specifically, we used the 3D positions of skeleton joints upon removing those that were assumed to “be occluded”, per case. The skeleton that occurred upon reconstruction was used to classify it into one of the predefined activities. To the best of our knowledge, this work was the first to treat the occlusion of moving body parts (i.e., subsets of skeleton joints) as a regression task. It has been demonstrated that the regression of missing joints could significantly improve the accuracy of classification. Particularly, it has been shown that a significant improvement of classification performance may be achieved by using the artificially reconstructed skeleton samples, rather than those affected by occlusion. This has been observed for any occlusion case and for almost any activity.

The novelty of this work is the reconstruction of the occluded data by treating this problem as a Generative Adversarial Network (GAN) task. Specifically, a GAN methodology is employed and various cases of partial occlusion are examined, i.e., occlusion of (a) an arm; (b) a leg; (c) both arms; (d) both legs; and (e) an arm and a leg on the same side. A Convolutional Recurrent Neural Network (CRNN) is trained to serve as the generator of the GAN, with the aim of producing a full skeleton by completing the missing parts. The input to this CRNN consists of raw 3D skeleton joint positions, but joints corresponding to the occluded parts have been removed. The output of the CRNN is a reconstructed skeleton, which is then fed into a long short-term memory (LSTM) network for classification into one of the predefined activities. For the discriminator, a simple long short-term memory (LSTM) network is used. Note that a separate network per occlusion case is trained and the effectiveness of the presented approach is evaluated by using four publicly available human activity recognition datasets and compared to a rigorous evaluation protocol, proposed in previous work [8]. As far as we know, the presented study is the first to address the occlusion of moving body parts (i.e., specific subsets of skeleton joints) within the framework of a GAN-based approach.

The rest of this paper is organized as follows: In Section 2, research works that deal with the effect of occlusion in HAR-related scenarios are presented. Then, in Section 3, the proposed reconstruction methodology is presented. Experimental results are presented in Section 3.5 and discussed in Section 5, wherein plans for future work are also presented. Finally, conclusions are drawn in Section 6.

## 2. Related Work

As previously noted, Human Activity Recognition (HAR) is among the most challenging research areas in computer vision, leading to a significant amount of research in this field in recent years [1]. Most of these works are based on 2D representations of skeletal motion [9,10,11,12,13,14]. Although it is both widely acknowledged and intuitive that occlusion significantly compromises the performance of HAR approaches [15], there exist only a few studies that specifically examine its impact on HAR performance or propose methods to mitigate it. In previous work [7], we have studied the effect of occlusion in 3D skeleton data. Specifically, it was simulated by removing body parts during the whole duration of the activity, and experimentally it was proved that the removal of one or both arms led to a significant drop of performance, in case of activities that are typically expressed by arm motion.

Several research works have dealt with occlusion. Specifically, Angelini et al. [16] generated synthetic partially occluded skeletons upon manually removing specific joints, per case, and showed that an increase to the recognition performance could be achieved by incorporating such occluded samples within the training procedure. Similarly, Iosifidis et al. [15] utilized a multi-camera configuration, encircling the subject, and simulated occlusion using the assumption that not all cameras can concurrently record skeleton motion. Moreover, recognition was based on fusion using solely cameras that remained “unaffected” by any case of occlusion. Moreover, Li et al. [17] used a bag of 3D points to depict poses and simulated occlusion by rejecting 3D points located in areas deemed to be affected by occlusion. Kim et al. [18] addressed the challenge of skeleton-based action recognition in occlusion scenarios by leveraging an occluded part detector and optimal joint group selection based on skeletal symmetry and angular information. In a similar manner, Chen et al. [19] introduced a Part-aware and Dual-inhibition Graph Convolutional Network (PDGCN) alongside a novel Dual Inhibition Training strategy aimed at enhancing skeleton-based action recognition in conditions of occlusion and noise. This is achieved by incorporating modules designed for occlusion simulation, global and local representation learning, and the gradual fusion of features. Gu et al. [20] generated occlusion masks, which were then utilized in both training and evaluation. In that case, a regression network was employed to reconstruct the missing skeleton parts. A single-pose embedding network was pre-trained in the work of Yang et al. [21]. The network’s goal was to learn occlusion-robust representations of pose sequences. The authors used an encoder to create the pose embeddings, and also a contrastive module to render this space occlusion invariant. Ma et al [22] also worked on the problem of human pose transfer by using a Flow-based Dual Attention (FDA) GAN, addressing occlusion and deformation in feature fusion for pose generation. Their model incorporated deformable local attention and flow similarity attention, so as to handle spatial correlations. Hernandez Ruiz et al. [23] proposed a bidirectional GAN framework, namely “GAN-poser”, for human motion prediction, able to handle occlusion, by learning to fill missing body parts with “plausible” motion patterns. Specifically, they enhanced the traditional GAN framework by including both forward and backward pathways, allowing better modeling of sequence dependencies and improving prediction quality. Moreover, Lee et al [24] proposed a Denoising Graph Autoencoder (DGAE) model to address the issue of missing joints in skeleton-based human action recognition (HAR) caused by occlusion and invisibility. By reconstructing missing joint coordinates with minimal error, the proposed model enhances the performance of HAR through the use of a masking Laplacian matrix to adjust feature weights and a Laplacian matrix in the decoder for reconstruction.

On the other hand, GANs have been widely used in several sensor-based HAR tasks. Irsch et al. [25] analyzed unlabeled data from wearable sensors to address data scarcity. Similarly, Li et al. [26] used GANs for sensor data augmentation. Abedin et al. [27] explored adversarial knowledge transfer for sequential sensor data. Rahman et al. [28] addressed kinematic inconsistencies in synthetic data using physics-aware models to enhance realism. Soleimani et al. [29] used GANs for cross-subject transfer learning and Jimale et al. [30] used GANs to produce synthetic data that are able to more accurately represent real data. Further, Qu et al. [31] compared the effects of GAN-generated synthetic spectrograms on classification accuracy. Wang et al. [32] explored a generative adversarial framework for sensor data generation, and Gammulle et al. [33] discussed a multi-level sequence GAN model for recognizing group activities by learning intermediate representations. Erol et al. [34] explored the use of GANs for augmenting radar-based data, improving the accuracy and robustness of HAR systems. Zadeh et al. [35] employed GANs to generate additional training data, while Wang et al. [36] leveraged GANs to combine information from multiple sensor data streams. Hasan et al. [37] introduced an innovative framework for gait recognition that tackles occlusion issues. This approach integrates an Occlusion Detection and Reconstruction (ODR) component based on 3D generative adversarial networks, along with a Feature Extraction for Gait Recognition (FEGR) module that employs both 3D and 2D CNNs to capture partwise and complete body features. Xu et al. [38] developed a technique named ActFormer, which is a GAN-based Transformer architecture designed for generating 3D human motion conditioned on actions, capable of managing both individual and multi-person interactive actions.

In the case of activity recognition using visual motion data, GANs are typically applied so as to generate realistic synthetic data to augment training datasets and/or enhance motion data quality, thereby improving the robustness and accuracy of activity recognition models in real-life scenarios. Various studies that are based on the use of 3D skeletal motion data have demonstrated the significant impact of GANs on HAR through data augmentation, transfer learning, and synthetic data generation. Fukushi et al. [39] proposed a few-shot generative model for skeleton-based human actions that leverages large source datasets to augment limited target domain samples using cross-domain and entropy regularization losses. Song et al. [40] addressed the challenge of recognizing human actions from noisy skeleton data by proposing a noise adaptation scheme based on GANs to handle noisy skeletons. Specifically, their model adapts features from noisy skeletons to a low-noise space using adversarial learning. Zalapeda et al. [41] proposed the use of synthetic data to augment the training dataset. They employed a GAN architecture to generate synthetic skeleton data and investigated the effect of different proportions of synthetic data in the training set. Degardin et al. [42] introduced Kinetic-GAN, i.e., an architecture combining GANs and Graph Convolutional Networks (GCNs) to generate realistic human body kinetics while maintaining spatial and temporal coherence. Avola et al. [43] proposed an approach for data augmentation, wherein Continuous Recurrent Neural Networks with GANs (C-RNN-GANs) were used to generate suitable synthetic action sequences to ensure robustness in cases such as incorrect skeleton extraction, perspective changes, and partial body occlusions. Shen et al. [44] addressed the challenge of limited training data in skeleton-based recognition and proposed an architecture which automatically approximates the distribution of input data and generates synthetic data, namely Imaginative GAN (Im-GAN), able to generate realistic and diverse synthetic data. Moreover, Pan et al. [45] presented a view normalization-based recognition framework to address viewpoint variance. They proposed an architecture, namely VN-GAN, wherein the generator estimates transformation parameters, applying them to normalize the viewpoint of input skeleton sequences. Liu et al. [46] proposed a generative network to generate high-quality human action data using motion style transfer and active learning. Finally, Li et al. [47] used manually defined occlusion areas, transforming a skeleton into a feature matrix. An attention model was integrated into a GAN to complete missing data of the aforementioned feature matrix.

Despite significant progress in HAR, several limitations persist, particularly regarding the challenge of occlusion. While the impact of occlusion on HAR performance is well-known, only a few studies have specifically addressed this issue, often relying on simplified scenarios where occlusion is simulated by manually removing specific joints or body parts. Such approaches may not fully capture the complexity of real-world occlusions. Additionally, methods like those proposed by Iosifidis et al. [15] depend on multi-camera configurations, which, while effective, are impractical for many real-world applications due to their cost and setup requirements. Moreover, many models are evaluated on controlled datasets with predefined occlusion conditions, limiting their generalizability to diverse or dynamic real-world environments. Reconstruction-based methods, such as those by Gu et al. [20] and Hernandez Ruiz et al. [23], have shown promise, but reconstructed joints often fail to accurately replicate real motion patterns, potentially affecting recognition performance. Advanced architectures like FDA-GAN and Denoising Graph Autoencoders (DGAEs) address occlusion with high computational demands, which can hinder real-time deployment. Furthermore, reliance on synthetic data or simulations, as seen in works like those by Yang et al. [21] and Chen et al. [19], raises concerns about their applicability in real-world scenarios. Addressing these limitations, the proposed approach focuses on developing a more robust, efficient, and generalizable approach to tackle occlusion in HAR.

## 3. Materials and Methods

### 3.1. Skeletal Data and Occlusion

#### 3.1.1. Occlusion of Skeletal Data

**Figure 1:**
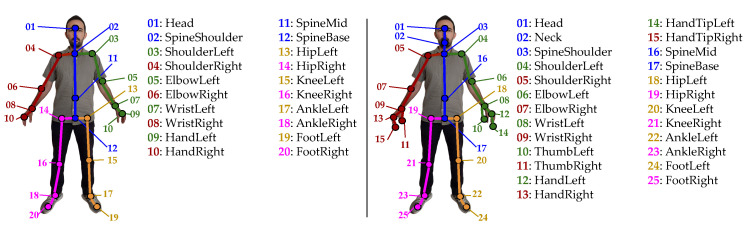
A human body pose with the 20 and 25 skeletal joints that are extracted using the Microsoft Kinect v1 (**left**) and v2 (**right**) cameras. Joints have been divided into subsets, each corresponding to one of the five main body parts, i.e., torso (blue), left hand (green), right hand (red), left leg (orange), and right leg (magenta). For illustrative purposes and also to facilitate comparisons between the two different versions, body parts have been colored using the same colors. Numbering follows the Kinect SDK in both cases; therefore, there exist several differences between the two versions.

Similarly to previous research efforts [2,48,49,50,51,52,53], the herein presented approach is also based on 3D trajectories of human skeletons that have been captured using the Kinect v1/v2 camera, comprising 20 or 25 joints, respectively. In Figure 1 a skeleton extracted using Kinect v1 and v2, with joints grouped to form distinct body parts, is presented.

As already mentioned in Section 1, partial occlusion may compromise the performance of HAR in real-life scenarios. Within the context of several applications, such as ambient assisted environments, AR environments, etc., occlusion typically occurs due to, e.g., activities taking place behind furniture, or due to the presence of more than one person in the same room. Of course, it should be obvious that the effects of occlusion vary depending on the activity performed. For example, the occlusion of both legs when the subject performs the action “kicking” results in a significant loss of visual information, which in turn may result to failure of recognition, while the occlusion of both arms is not expected to compromise recognition for this activity. Although the aforementioned example is quite extreme, it is common sense that partial occlusion may hinder the overall effectiveness of HAR approaches.

#### 3.1.2. Datasets

We are not aware of any publicly available datasets that contain real 3D occluded actions. To address this and in order to evaluate the proposed methodology, structured subsets of skeletal joints forming body parts (e.g., arms and legs) have been manually excluded from four publicly available datasets that provide 3D skeletal information. Specifically, these datasets are summarized in Table 1 and may be briefly described as follows:

- The PKU-MMD dataset [54] is a publicly available and open-source benchmark for 3D human motion-based activity recognition. From this dataset, we opted for 11 actions that are tightly related to activities of daily living (ADLs) [51,55], i.e., eating, falling, handshaking, hugging, making a phone call, playing with a phone or tablet, reading, sitting down, standing up, typing on a keyboard, and wearing a jacket, which correspond to 21,456 data samples.
- The NTU-RGB+D dataset [56] is also a large-scale benchmark for 3D human activity analysis. From this dataset, we opted for a subset consisting of medical conditions, which includes 12 classes and 11,400 samples, i.e., sneezing/coughing, staggering, falling, headache, chest pain, back pain, neck pain, nausea/vomiting, fanning oneself, yawning, stretching, and blowing one’s nose.
- SYSU 3D Human–Object Interaction (HOI) [57] is a dataset that focuses on 3D human motion-based interactions between people and objects. It contains 480 activity samples from 12 different activities, i.e., drinking, pouring, calling a phone, playing with a phone, wearing backpacks, packing backpacks, sitting on a chair, moving a chair, taking out a wallet, taking from a wallet, mopping, and sweeping. Within the aforementioned activities, 40 subjects and one of the following objects per case were involved: phone, chair, bag, wallet, mop, and besom. Each activity has 40 samples.
- The UTKinect-Action3D dataset [58] includes 10 different activities that were performed by 10 different subjects, i.e., walking, sitting down, standing up, picking up, carrying, throwing, pushing, pulling, waving hands, and clapping hands. Each activity was performed twice by each subject, resulting in a total of 200 activity instances.

From the aforementioned datasets, we only used 3D skeleton motion data and disregarded other modalities. Also, PKU-MMD and NTU-RGB were recorded using Microsoft Kinect v2 under three camera viewpoints, while SYSU-3D-HOI and UTKinect-Action3D were recorded using Microsoft Kinect v1 under a single camera viewpoint.

**Table 1:** Summary of datasets for human activity recognition (HAR) that have been used for the experimental evaluation of this work.

Name	Activities	Participants	Examples	Types of Activities
PKU-MMD [54]	51	66	∼20,000	Daily, sports, and health-related activities
NTU RGB+D [56]	60	40	∼56,000	Daily, interactive, and health-related actions
SYSU-3D-HOI [57]	12	40	480	Human–Object Interactions
UTKinect-Action-3D [58]	10	10	200	Interactive and gesture-based actions

**Figure 2:**
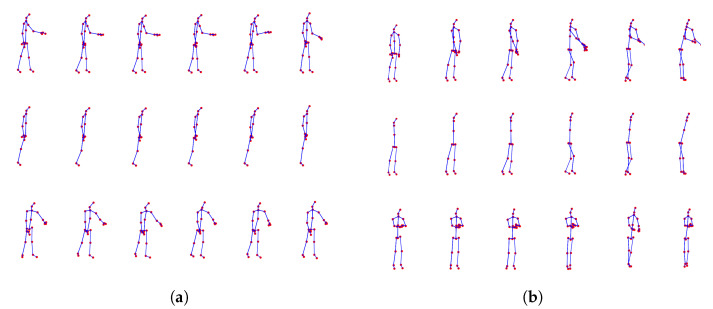
Example skeleton sequences of the activities (**a**) *handshaking* and (**b**) *hugging other person* from the PKU-MMD dataset, captured by Microsoft Kinect v2. First row: original skeletons, including all 25 joints (i.e., without any occlusion); second row: joints corresponding to (**a**) left arm; (**b**) both arms (see Figure 1) have been discarded (i.e., the skeleton is partially occluded); third row: skeletons have been reconstructed using the proposed deep regression approach. The example of (**a**) is successfully reconstructed and correctly classified, while the example of (**b**) is unsuccessfully reconstructed and incorrectly classified.

It should be noted that most public motion-based datasets, such as the ones herein used for evaluation purposes, have been created under ideal laboratory conditions, and thus occlusion is prevented. Since the creation of a large-scale dataset is a time-consuming task, it was decided to follow an approach such as the one of [7,8,20], i.e., subsets of joints that correspond to body parts will be manually discarded, assuming that these parts remain occluded during the whole duration of each activity (see Figure 1). Moreover, and for the sake of explanation, two visual examples of activities with/without occlusion are illustrated in Figure 2, where the loss of visual information is easily comprehensible. Specifically, an example of a successfully reconstructed skeleton, which leads to correct classification, and another example of an unsuccessfully reconstructed skeleton, which leads to incorrect classification, are both therein illustrated.

### 3.2. Generative Adversarial Networks

A generative adversarial network (GAN) [59] is a framework for machine learning, mainly used in generative AI [60]. In brief, it consists of two neural networks that take part in a game. Within this game, the gain of one network leads to a loss of the other. The one network is the generator, while the other is the discriminator. The GAN aims to learn how to generate “new” examples which follow the same statistics as those that have been used for training. For example, in the presented approach, a GAN trained on skeleton sequences shall be used; the goal of its generator will be to generate new skeleton sequences that will be “realistic” compared to the real ones that have been used for training. This is achieved based on training through feedback by the discriminator, which is able to discriminate between “realistic” and “non-realistic” images. Both generator and discriminator are dynamically updated, i.e., they are trained in a way that the the former learns to “fool” the latter. Specifically, a given sample that is generated by the generator should be mapped to a specific distribution; each of them should be recognized as belonging to the true distribution or not by the discriminator.

#### 3.2.1. Generator

**Figure 3:**
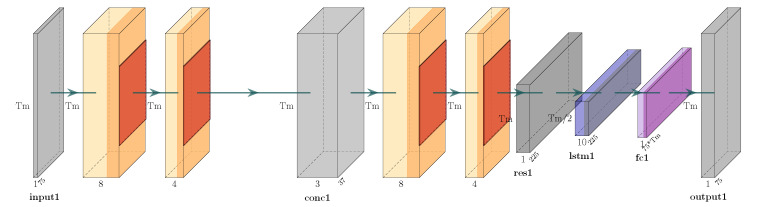
The architecture of the generator of the proposed GAN.

**Figure 4:**
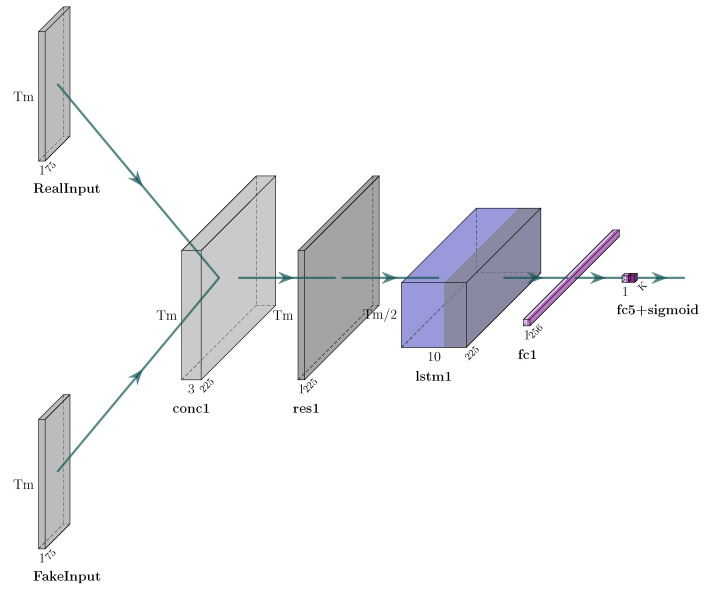
The architecture of the discriminator of the proposed GAN architecture.

The generator is formulated as a *regression* task, i.e., the one presented in [8]. Specifically, let X denote the original skeleton sequence and $X_{o}$ the sequence resulting from occlusion. The aim of regression, i.e., of the generator, is to estimate a set of parameters β of a given function *f*, so that $X=f(X_{o},\beta )+\epsilon$, where ϵ is some error value, can be minimized. The Convolutional Recurrent Neural Network (CRNN) model of previous work [8] is employed as the generator model for the GAN framework to achieve the objective. Therefore, the purpose of this model is to execute *f* and learn β (i.e., its weights) in order to reduce ϵ. When given an occluded skeleton sequence $X_{o}$, the network generates a reconstructed skeleton sequence $X_{r}$, which is an approximation of X, by filling in the missing (occluded) data (joints). Also, in this case, the CRNN includes an LSTM whose goal is to capture temporal information of skeletal data. Figure 3 and Figure 4 illustrate the architectures of the CRNN and the LSTM networks, respectively.

#### 3.2.2. Discriminator

The discriminator uses as input a generated image and a real image and reshapes them according to the timeframes that have been set. Its architecture is quite simple: it first feeds the aforementioned images into an LSTM layer, followed by two dense layers, the first comprising 256 neurons and the last solely 1 neuron that produces a binary result, i.e., to determine whether the image is real or not. The discriminator loss is a sigmoid cross-entropy loss of the real and generated images. The architecture of the discriminator is illustrated in Figure 4.

### 3.3. Classification

**Figure 5:**
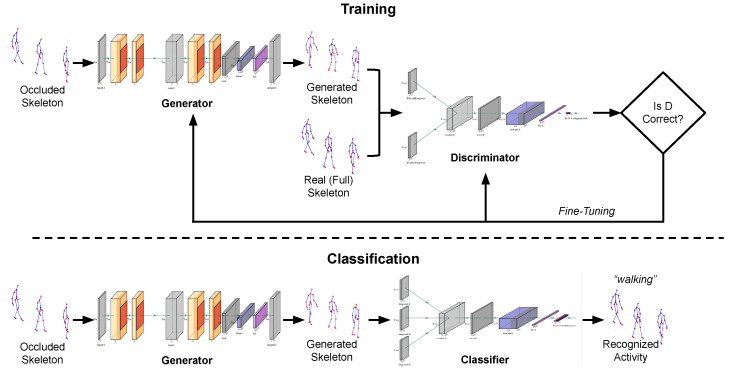
A visual overview of the proposed approach.

An occluded activity sample (i.e., a sample with missing body parts resulting from occlusion) is fed as input to the trained CRNN network, whose role is to reconstruct the missing skeletal data. For the sake of explanation, two visual examples of an activity before and after reconstruction are shown in Figure 2. Upon reconstruction, the proposed approach proceeds with the classification of this sample into one of the predefined activities. For classification, a LSTM network with 1 layer is used. It should be herein emphasized that the CRNN network is trained using only *full skeletal data* X, i.e., not affected by occlusion of any body part(s). Conversely, during evaluation, the input to the LSTM network consists of the corresponding skeletal sequences $X_{r}$, which have been reconstructed from X. A visual overview of the proposed approach is illustrated in Figure 5.

**Figure 6:**
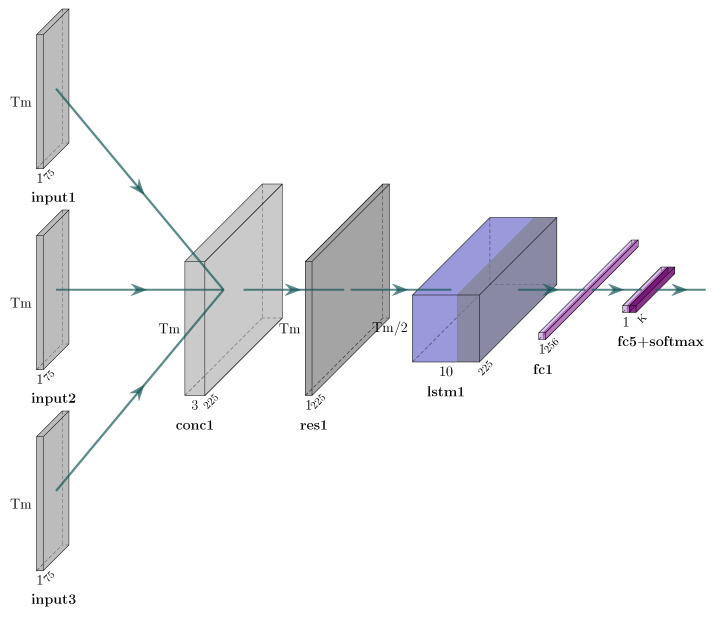
The architecture of the classifier of the proposed approach for the three-camera case.

**Figure 7:**
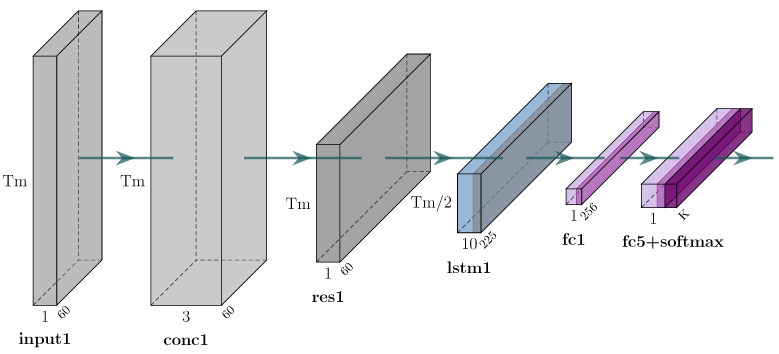
The architecture of the classifier of the proposed approach for the one-camera case.

To assess the performance of the herein proposed skeleton reconstruction approach, a classifier to measure the accuracy of the model is used. The architecture of the classifier is illustrated in Figure 6. Specifically, in case wherein three different camera viewpoints are used, the input from all cameras are combined into one, reshaped to fit the predetermined timeframes, and then fed into an LSTM layer. Following this step, two dense layers are used, the first comprising 256 neurons and the last comprising 1 neuron that produces a result which is an indication of the user’s activity (see Figure 6). For datasets with a single camera viewpoint, the same network is used, hence with only one input (see Figure 7).

### 3.4. The GAN Objective

In this work, the objective of the Pix2Pix GAN framework [61] is adopted. Specifically, the objective $L_{\mathrm{cGAN}}$ of a conditional Generative Adversarial Network (GAN) is described by the following:

(1) $$L_{\mathrm{cGAN}}\left(G,D\right)=E_{x,y}\left[\log D\left(x,y\right)\right]+E_{x,z}\left[\log\left(1-D\left(x,G\left(x,z\right)\right)\right)\right].$$

Previous approaches have suggested combining the GAN objective with a more traditional loss, such as the L1 distance; in that case, it should be “close” to the real output:

(2) $$L_{L1}\left(G\right)=E_{x,y,z}{[∥y-G\left(x,z\right)∥}_{1}]$$

Thus, upon combination, the final objective is

(3) $$G^{*}=\arg\min_{G}\max_{D}L_{cGAN}\left(G,D\right)+\lambda L_{L1}\left(G\right)$$

### 3.5. Experiments

#### 3.5.1. Experimental Setup and Network Training

Experiments were conducted on a personal workstation with an Intel™i7 4770 4-core processor running at 3.40 GHz and 32 GB RAM, using an NVIDIA™Geforce RTX 3070 GPU with 8 GB VRAM and Ubuntu 20.04 (64 bit). The deep architecture was implemented in Python using Keras 2.4.3 [62] with the Tensorflow 2.5 [63] backend. All data pre-processing and processing steps were implemented in Python 3.9 using NumPy and SciPy. For the training of the generator, the LeakyReLU activation function was used, except for the LSTM layer where the tanh function was used, and the last dense layer where the linear activation function was used. For the training of the classifier, the LeakyReLU and tanh activation functions were used, respectively, except for the last layer, where the sigmoid activation function was used. The batch size was set to 5 and 10 for the training of the classifier and the GAN, respectively. The Adam optimizer was utilized in both cases, the dropout was configured to 0.25, the learning rate to 0.001, and the training was conducted for 200 epochs, using the loss of the validation set calculated via MSE as an early stopping method to prevent overfitting. In all cases, 80% of the available activity samples was used for training, 10% for validation, and the remaining 10% for evaluation.

As in previous work [8], the herein presented approach takes temporal sequences of 3D skeleton data as input, while interpolation is applied to set the duration of all activity samples equal to $T_{m}$, which is the size of the sample of max duration. Note that experiments consider both single- and multi-view datasets. In the latter case, all available views are utilized by feeding the network with all three skeleton sequences. It is also assumed that occlusion affects the same body part(s), in every view, and within the whole duration of the activity. The presented approach is based on the idea that since occlusion leads to missing body parts (i.e., in this case some of the skeleton joints have been discarded, so the respective coordinates are missing), the problem of “reconstructing” the respective values of missing body parts could be devised as a generative adversarial network (GAN) task. Again, as in [8], one network per occlusion case is trained, resulting in eight different networks. Thus, by giving a sequence of skeletal data as input, while some action is being performed, the missing skeletal joints are initially identified and then fed to the appropriate trained network, based on the corresponding occlusion case. Then, using this network, it is classified into an activity class. Note that even though the proposed approach requires more memory to store all the aforementioned networks, this is compensated by the significant increase in performance compared to the use of a single network. It should be emphasized that all the classifiers are trained exclusively using samples not affected by occlusion.

At this stage, a sample with missing skeletal joints due to occlusion can be used as input into the trained generator, which then reconstructs the missing skeletal data. An example of this activity before and after reconstruction is shown in Figure 2. After reconstruction, the data can be classified into one of the pre-defined classes using the classifier. During the testing phase, the input of the classifier is a reconstructed skeletal sequence $X_{r}$.

#### 3.5.2. Evaluation Protocol

To experimentally evaluate the proposed methodology, the following series of experiments is considered:

- Removal of structured sets of skeletal joints, corresponding to body parts, to simulate occlusion (see Figure 1). Specifically, as already mentioned, cases of part removal include (a) left arm; (b) right arm; (c) both arms; (d) left leg; (e) right leg; (f) both legs; (g) left arm and left leg; (h) right arm and right leg. We used an LSTM network that had been trained using exclusively samples that were not affected by occlusion, and also the skeletons were reconstructed using a GAN;
- A “baseline” approach, where both training and evaluation of the LSTM took place using exclusively samples not affected by occlusion;
- A “reference” approach where training of the LSTM took place using exclusively samples not affected by occlusion, but was evaluated using occluded samples;
- An approach wherein samples affected by occlusion were included in the training process of the LSTM, while validation was performed exclusively using occluded samples. Here, a subset equal to 10% of the non-occluded samples of the training set was selected. From these samples, all eight cases of occlusion have been generated, thus “augmenting” the initial training data by 80%. Note that a single network was used for all eight cases of occlusion.

Note that in this study, we strictly adhere to the predefined evaluation protocols associated with each dataset, which dictate a single training and testing procedure on fixed splits, so as to ensure comparability of results.

## 4. Results

**Figure 8:**
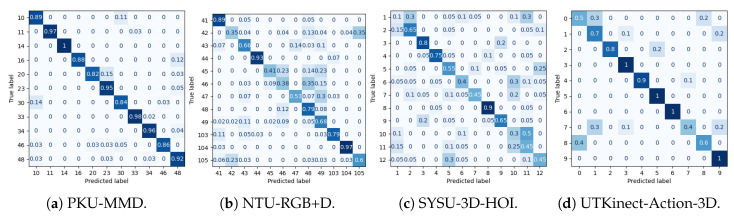
Normalized confusion matrices for classification for all datasets, without removing any body part.

**Figure 9:**
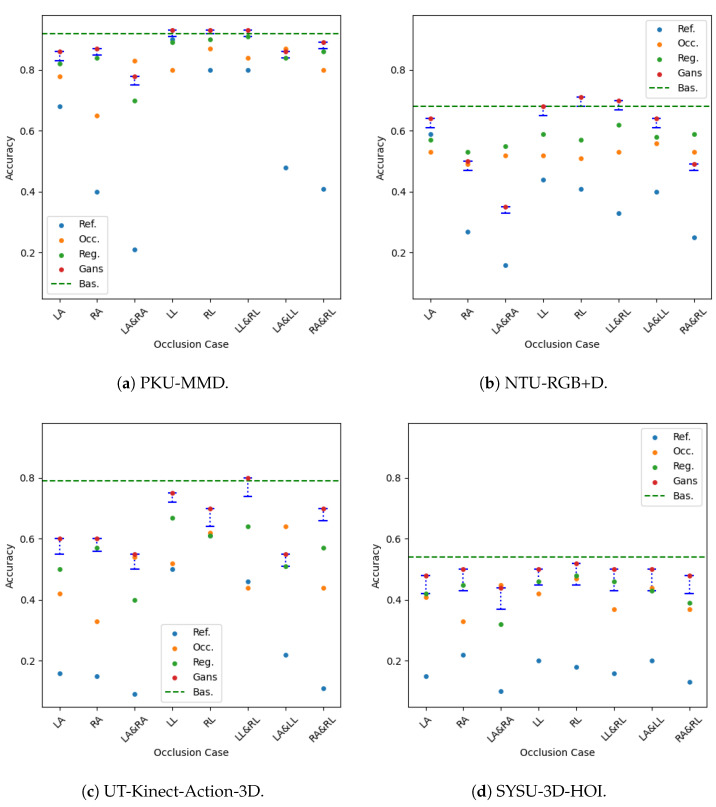
Confidence intervals using the proposed approach on all datasets, compared with the best weighted accuracies reported in previous works. In case of the proposed approach, red dot denotes the upper bound of the confidence interval, i.e., the best weighted accuracy achieved.

**Figure 10:**
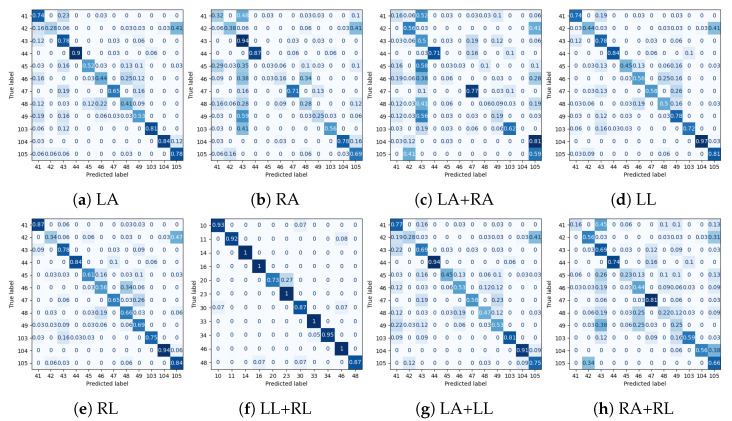
Normalized confusion matrices for classification for the NTU-RGB+D dataset. LA, RA, LL and RL correspond to cases of occluded Left Arm, Right Arm, Left Leg and Right Leg, respectively.

**Figure 11:**
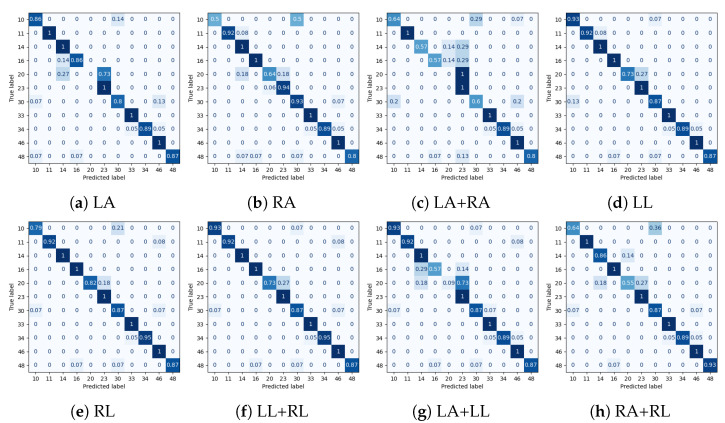
Normalized confusion matrices for classification for the PKU-MMD dataset. LA, RA, LL and RL correspond to cases of occluded Left Arm, Right Arm, Left Leg and Right Leg, respectively.

**Figure 12:**
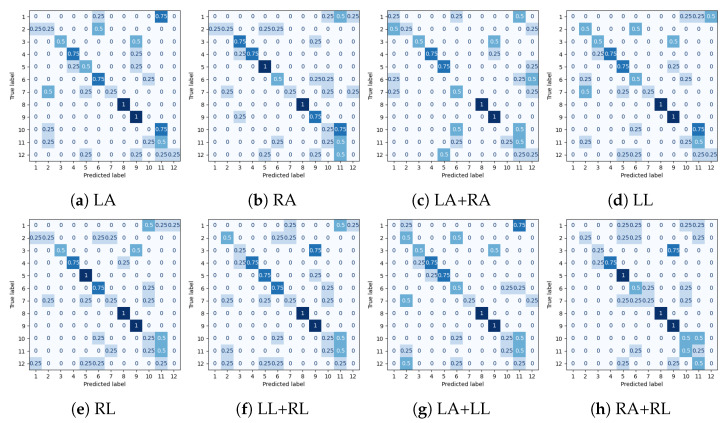
Normalized confusion matrices for classification for the SYSU-3D-HOI dataset. LA, RA, LL and RL correspond to cases of occluded Left Arm, Right Arm, Left Leg and Right Leg, respectively.

**Figure 13:**
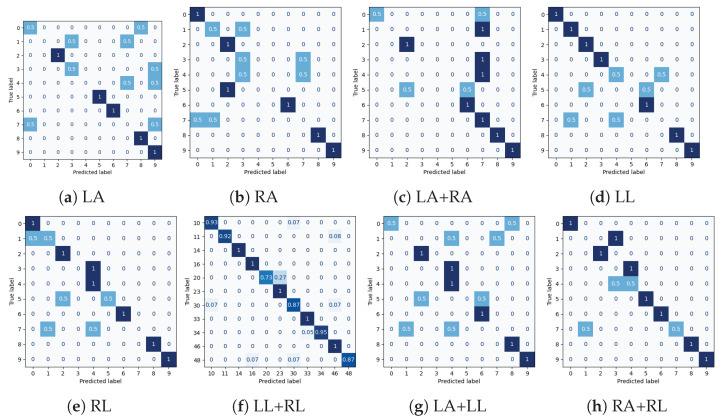
Normalized confusion matrices for classification for the UT-Kinect-Action-3D dataset. LA, RA, LL and RL correspond to cases of occluded Left Arm, Right Arm, Left Leg and Right Leg, respectively.

Experimental results for all datasets are depicted in Table 2, Table 3, Table 4 and Table 5, while most extensive results, per class and per occlusion case, are depicted in the Appendix and specifically in Table A1, Table A2, Table A3 and Table A4. The following metrics have been extracted: accuracy per class, and F_1_-score per class and weighted accuracy, where class weights were calculated based on the class distribution. Moreover, confusion matrices for all datasets in the case of the baseline experiment are depicted in Figure 8. Note that in Table 2, Table 3, Table 4 and Table 5 and in Figure 9, confidence intervals of the proposed approach are presented and compared to the best results reported in previous works. Furthermore, the case of the classification of reconstructed samples is depicted in Figure 10, Figure 11, Figure 12 and Figure 13. We also performed comparisons using the aforementioned protocol with two previous works. Specifically, in [64], a data augmentation approach was presented, wherein a CNN was trained with the addition of artificially occluded samples in the training set, while in [8], a deep regression approach for skeleton reconstruction was presented. Note that although the same evaluation protocol and the same datasets are used as in these works, the herein presented work formulates the problem of reconstruction as a GAN task, and is hence based on a novel GAN architecture.

In the case of the PKU-MMD dataset, the weighted accuracy (WA) was 0.92 without any body part removal. Specifically, it ranged between 0.21 and 0.90 in case of some body part removal, while it ranged between 0.78 and 0.93 upon reconstruction with the GAN. In all cases, significant improvement was observed in terms of WA. Moreover, reconstruction with the GAN outperformed the regression approach [8] in all cases, and the augmentation approach [64] in 6 out of 8 cases. Since most of the activities used to evaluate our approach primarily involve upper body motion (i.e., are expressed by the motion of left and/or right arm), this is also reflected to the results of Table A1, wherein it may be observed that in cases of occluded arms, the improvement is significantly large, with the most notable example being the case of both arms, wherein WA improves from 0.21 to 0.78. Upon careful observation of the confusion matrices depicted in Figure 11, for each occlusion case, the following should be noticed when comparing the case where all joints were used:

- In the case of any occluded arm, class *make a phone call/answer phone* is often confused with *playing with phone/tablet*. In the case of the occluded right arm, class *eat meal/snack* is very often confused with *reading*. This happens less often in the case of the occluded left arm. Also, in a few cases, class *handshaking* is confused with *hugging*, and class *reading* with *typing on a keyboard*.
- In the case of any occluded leg, class *make a phone call/answer phone* is often confused with *playing with phone/tablet*. Also, in the case of occluded left leg, class *reading* is often confused with *eat meal/snack*, while in the case of occluded right leg, class *eat meal/snack* is very often confused with *reading*.
- In the case of occluded left arm and left leg, class *make a phone call/answer phone* in the majority of testing examples is confused with *playing with phone/tablet* and class *hugging* with *playing with phone/tablet* or *handshaking*.
- In the case of occluded right arm and right leg, class *make a phone call/answer phone* is often confused with *playing with phone/tablet* or *handshaking*, *eat meal/snack* is very often confused with *reading*, and class *handshaking* is often confused with *make a phone call/answer phone*.

In all cases, the majority of classes demonstrate excellent performance, equivalent to the case without any occlusion. Compared to the augmentation approach of [64], it should be noted that reconstruction using GANs was superior in 6 out of the 8 cases of occlusion. Specifically, augmentation showed superior performance in the case of both occluded arms and in the case of occluded left arm and left leg, although in that case, reconstruction using GANs exhibited equivalent performance. Finally, as shown in Figure 9a, in most cases, the lower bound of the weighted accuracy’s confidence interval exceeds a better performance than all other approaches.

In the case of the NTU-RGB+D dataset, the WA was 0.68 without any body part removal and ranged between 0.16 and 0.59 in the case of some body part removal, while it ranged between 0.35 and 0.71 upon reconstruction with the GAN. In that case, in 5 out of 8 cases, significant improvement was observed in terms of WA, while performance was almost equal with the other two approaches in the case of removal of right arm or right arm and right leg. In the remaining three cases, the regression approach demonstrated the best performance. Moreover, reconstruction with GAN outperformed the augmentation approach in 7 out of 8 cases. Since activities used to evaluate the proposed approach mainly consisted of upper body motion, also in the case of the NTU-RGB+D dataset, in the results of Table A2, it could be observed that in all the remaining cases of occluded arms, the improvement of WA is large, with the most notable example being the case of both arms, wherein WA improves from 0.16 to 0.35, although the other two approaches demonstrated superior performance in that case. Upon careful observation of the confusion matrices depicted in Figure 10, and considering the classification results without occlusion of any part, for each occlusion case it should be noticed that most activities are affected by the occlusion of the right arm and performance is far from being excellent in the majority of these cases. Notably, reconstruction with GANs exhibits best performance in case of occluded legs. Finally, also in this dataset and as shown in Figure 9b, in most cases, the lower bound of the weighted accuracy’s confidence interval exceeds best performance of all other approaches.

In the case of the SYSU-3D-HOI dataset, the WA was 0.54 without any body part removal and ranged between 0.10 and 0.22 in the case of some body part removal, while it ranged between 0.44 and 0.52 upon reconstruction with the GAN, as may be observed in the results of Table A4. Moreover, the reconstruction with GANs outperformed the other two methods in 7 out of 8 cases. In every case of occlusion, significant improvement was observed in terms of WA when compared to the reference case. It should be noticed that due to the small size of this dataset, performance was inadequate in several classes even without occlusion, while reconstruction in several cases exhibited superior performance. It was quite a surprise to observe that reconstruction with GANs steadily exhibited almost constant performance for all occlusion cases, whilst in many occasions it was also superior to the baseline approach, i.e., to the absence of occlusion. Also, due to the small size of the dataset, often several classes fail to be recognized. Finally, also in this dataset and as shown in Figure 9b, in several cases, the lower bound of the weighted accuracy’s confidence interval does not exceed best performance of all other approaches; however, this was expected due to the small dataset size.

In the case of the UTKinect-Action-3D dataset, the WA was 0.79 without body part removal, and ranged between 0.09 and 0.61 in the case of some body part removal and also between 0.55 and 0.80 upon reconstruction with GAN. Moreover, reconstruction with GANs outperformed the other two methods in 7 out of 8 cases. Since activities used to evaluate the proposed approach mainly consisted of upper body motion, also in the case of the UTKinect-Action-3D dataset, in the results of Table A3 it could be observed that in all the remaining cases of occluded arms, the improvement of WA is exceptional, with the most notable examples the cases of occluded right arm and right leg, wherein WA improves from 0.11 to 0.70 and right arm and left arm wherein WA improves from 0.09 to 0.55. Upon careful observation of the confusion matrices depicted in Figure 13 and considering the classification results without occlusion of any part, for each occlusion case we should notice that occlusion affects half of the activities, especially those that are based on the motion of arms. Finally, also in this dataset and as shown in Figure 9c, in most cases the lower bound of the weighted accuracy’s confidence interval exceeds best performance of all other approaches.

In all cases, the occlusion of some body parts leads to a severe drop in performance; in many cases, the accuracy and F_1_-score of some classes dropped to zero/near zero values. In some instances, the reconstruction was successful, resulting in nearly perfect recognition. However, even in these cases, the classification of reconstructed samples for several activities and specific occlusion scenarios showed slightly inferior performance compared to the occluded samples. We believe that in these cases, the occluded body part is less “relevant” for those activities. Nonetheless, there are instances where the reconstruction approach may fail, leading to misleading joint positions, as demonstrated in the example of Figure 2.

## 5. Discussion and Future Work

The results across all datasets demonstrate that the occlusion of body parts significantly impacts recognition performance, often causing a severe drop in accuracy and F_1_-scores, with some classes dropping to near-zero values. However, reconstruction using GANs consistently showed notable improvements, outperforming alternative approaches in most cases. Specifically, in datasets like PKU-MMD and UTKinect-Action-3D, the weighted accuracy improved dramatically in scenarios involving occluded arms, underscoring the effectiveness of GAN-based reconstruction in handling upper-body motion activities.

Despite these successes, certain limitations persist. For activities less dependent on the occluded body parts, reconstruction sometimes exhibited slightly inferior performance compared to the baseline, suggesting a diminished relevance of those parts for the specific activities. Additionally, while GANs excelled in mitigating the effects of occlusion, occasional reconstruction failures resulted in misleading joint positions, potentially affecting classification accuracy. These findings highlight both the potential and the challenges of leveraging GANs for robust activity recognition in occlusion scenarios.

Future research work may target various aspects of the HAR problem with a focus on occlusion. Our regression approach could be enhanced and improved by, for example, replacing the interpolation step currently used with a temporal augmentation approach similar to that of Kwon et al. [65]. Additionally, incorporating other state-of-the-art architectures, such as transformers [66], into the classification process could yield significant improvements. Moreover, the aspect of occlusion in HAR, including scenarios like temporally partial occlusion, warrants further investigation. Since handcrafted features have been experimentally shown to enhance the recognition performance of deep learning approaches [67], experimenting with other feature extraction methodologies based on the geometry and motion of skeletons, such as the approach proposed by Avola et al. [43], would be of great interest. Finally, we also believe that conducting real-life experiments in an assistive living environment would be extremely valuable.

## 6. Conclusions

In this paper, a methodology for reconstructing human skeleton sequences was developed by employing a generative adversarial network. The generator in this network was a convolutional recurrent neural network, which was trained to generate the complete skeleton by filling in the missing parts. The input to the generator consisted of the raw 3D positions of the skeleton joints, with the occluded joints removed. The output of the generator was a reconstructed skeleton, which was then passed through a long short-term memory network for classification into one of the predefined activities. For the discriminator, a simple long short-term memory network trained only on non-occluded samples was used. It was demonstrated that in all scenarios, the occlusion of certain body parts resulted in a significant decline in performance, although the reconstruction process achieved nearly perfect recognition in some cases. Through experimental evaluation, it has also been demonstrated that the proposed approach offers improved performance compared to previous methodologies, which varied between 2.2% and 37.5%, depending on the chosen dataset and the specific occlusion scenario.

To conclude, in real-life applications, i.e., in dynamic and real-life scenarios, human activity recognition from visual data faces several limitations when dealing with occlusion. Real-life occlusions are inherently unpredictable, caused by factors such as environmental obstacles, people entering or leaving the frame, and self-occlusion, making them difficult to accurately model in controlled studies. Furthermore, most HAR datasets are collected in constrained environments with limited variability and predefined activities, reducing their generalizability to diverse real-world conditions. Sensor limitations, such as restricted camera angles and the impact of environmental factors like lighting or background clutter, intensify the challenges of recognizing occluded actions in dynamic settings. Current models often depend on synthetic or static occlusion scenarios for training, which fail to capture the complexity of real-world occlusions, and their computational demands can hinder real-time deployment. Additionally, standard evaluation metrics may not reflect the nuances of occlusion in real-world applications, and overlapping activities can create further ambiguity in recognition. Finally, ethical and privacy concerns during data collection in public spaces limit the scope of real-world studies, while participant behavior under observation may introduce biases. Addressing these limitations requires the use of multi-sensor systems, dynamic dataset creation, advanced occlusion-handling models, and robust ethical frameworks for real-world applicability.

## Figures and Tables

**Table 2:** Results on PKU-MMD dataset. “Bas.”/“Gans”/“Reg.”/“Occ.”/“Ref.” denote baseline/Generative adversarial networks/ Regression/training with occluded samples/reference case. “None” denotes the case without occlusion. LA, RA, LL, RL denote the occlusion of left arm, right arm, left leg, right leg, respectively. Numbers denote Weighted Accuracy, numbers in bold indicate best performance between “Rec.”/“Occ.”/“Ref.”. In case of Gans, by “max” and “min” we denote the upper and the lower bounds of the confidence interval.

	None		LA	RA	LA+RA	LL	RL	LL+RL	LA+LL	RA+RL
Gans	-	max	**0.86**	**0.87**	0.78	**0.93**	**0.93**	**0.93**	0.86	**0.89**
		min	0.83	0.85	0.75	0.91	0.92	0.91	0.84	0.87
Reg.	-		0.82	0.84	0.70	0.89	0.90	0.91	0.84	0.86
Occ.	-		0.78	0.65	**0.83**	0.80	0.87	0.84	**0.87**	0.80
Ref.	-		0.68	0.40	0.21	0.90	0.80	0.80	0.48	0.41
Bas.	0.92	-	-	-	-	-	-	-	-	-

**Table 3:** Results on NTU-RGB+D dataset.“Bas.”/“Gan”/“Reg.”/“Occ.”/“Ref.” denote baseline/Generative adversarial networks/ Regression/training with occluded samples/reference case. “None” denotes the case without occlusion. LA, RA, LL, RL denote the occlusion of left arm, right arm, left leg, right leg, respectively. Numbers denote Weighted Accuracy, numbers in bold indicate best performance between “Rec.”/“Occ.”/“Ref.”. In case of Gans, by “max” and “min” we denote the upper and the lower bounds of the confidence interval.

	None		LA	RA	LA+RA	LL	RL	LL+RL	LA+LL	RA+RL
Gans	-	max	**0.64**	0.50	0.35	**0.68**	**0.71**	**0.70**	**0.64**	0.49
		min	0.61	0.47	0.33	0.65	0.68	0.67	0.61	0.47
Reg.	-		0.57	**0.53**	**0.55**	0.59	0.57	0.62	0.58	**0.59**
Occ.	-		0.53	0.49	0.52	0.52	0.51	0.53	0.56	0.53
Ref.	-		0.59	0.27	0.16	0.44	0.41	0.33	0.40	0.25
Bas.	0.68	-	-	-	-	-	-	-	-	-

**Table 4:** Results on UTKinect-Action3D dataset.“Bas.”/“Gan”/“Reg.”/“Occ.”/“Ref.” denote baseline/Generative adversarial networks/Regression/training with occluded samples/reference case. “None” denotes the case without occlusion. LA, RA, LL, RL denote the occlusion of left arm, right arm, left leg, right leg, respectively. Numbers denote Weighted Accuracy, numbers in bold indicate best performance between “Rec.”/“Occ.”/“Ref.”. In case of Gans, by “max” and “min” we denote the upper and the lower bounds of the confidence interval.

	None		LA	RA	LA+RA	LL	RL	LL+RL	LA+LL	RA+RL
Gans	-	max	**0.60**	**0.60**	**0.55**	**0.75**	**0.70**	**0.80**	0.55	**0.70**
		min	0.55	0.56	0.50	0.72	0.64	0.74	0.51	0.66
Reg.	-		0.50	0.57	0.40	0.67	0.61	0.64	0.51	0.57
Occ.	-		0.42	0.33	0.54	0.52	0.62	0.44	**0.64**	0.44
Ref.	-		0.16	0.15	0.09	0.50	0.61	0.46	0.22	0.11
Bas.	0.79	-	-	-	-	-	-	-	-	-

**Table 5:** Results on SYSU-3D-HOI dataset. “Bas.”/“Gan”/“Reg.”/“Occ.”/“Ref.” denote baseline/Generative adversarial networks/Regression/training with occluded samples/reference case. “None” denotes the case without occlusion. LA, RA, LL, RL denote the occlusion of left arm, right arm, left leg, right leg, respectively. Numbers denote Weighted Accuracy, numbers in bold indicate best performance between “Rec.”/“Occ.”/“Ref.”. In case of Gans, by “max” and “min” we denote the upper and the lower bounds of the confidence interval.

	None		LA	RA	LA+RA	LL	RL	LL+RL	LA+LL	RA+RL
Gans	-	max	**0.48**	**0.50**	0.44	**0.50**	**0.52**	**0.50**	**0.50**	**0.48**
		min	0.42	0.43	0.37	0.45	0.45	0.43	0.43	0.42
Reg.	-		0.42	0.45	0.32	0.46	0.48	0.46	0.43	0.39
Occ.	-		0.41	0.33	**0.45**	0.42	0.47	0.37	0.44	0.37
Ref.	-		0.15	0.22	0.10	0.20	0.18	0.16	0.20	0.13
Bas.	0.54	-	-	-	-	-	-	-	-	-

## Data Availability

The PKU-MMD dataset is available at https://www.icst.pku.edu.cn/struct/Projects/PKUMMD.html (accessed on 3 March 2025). The NTU-RGB+D dataset is available at https://rose1.ntu.edu.sg/dataset/actionRecognition/ (accessed on 3 March 2025). The UT-Kinect-3D dataset is available at https://cvrc.ece.utexas.edu/KinectDatasets/HOJ3D.html (accessed on 3 March 2025). The SYSU-3D-HOI dataset is available at https://www.isee-ai.cn/~hujianfang/ProjectJOULE.html (accessed on 3 March 2025).

## Abbreviations

The following abbreviations are used in this manuscript:

2D	2-Dimensional
3D	3-Dimensional
AR	Augmented Reality
CRNN	Convolutional Recurrent Neural Network
CsiGAN	Channel State Information Generative Adversarial Network
ExGANs	Exemplar GANs
FDA	Flow-based Dual Attention
GAN	Generative Adversarial Network
GPU	Graphics Processing Unit
HOI	Human–Object Interaction
HAR	Human Activity Recognition
LSTM	Long Short-Term Memory
MSE	Mean Squared Error
RNN	Recurrent Neural Network
WA	Weighted Accuracy

## Appendix A

**Table A1:** Results on PKU-MMD dataset. “Bas.”/“Gan”/“Reg.”/“Occ.”/“Ref.” denote baseline/Generative adversarial networks/ Regression/training with occluded samples/reference case, Acc, F_1_ and WA denote Accuracy, F_1_ score and Weighted Accuracy, respectively. “None” denotes the case without occlusion. LA, RA, LL, RL denote the occlusion of left arm, right arm, left leg, right leg, respectively. Numbers in bold indicate best performance between “Rec.”/“Occ.”/“Ref.”. Numbers in italics denote the corresponding classes, as follows: 10: eat meal/snack, 11: falling, 14: handshaking, 16: hugging other person, 20: make a phone call/answer phone, 23: playing with phone/tablet, 30: reading, 33: sitting down, 34: standing up, 46: typing on a keyboard, 48: wear jacket.

		None	LA				RA				LA+RA				LL				RL				LL+RL				LA+LL				RA+RL			
Class	Metric	Bas.	Gan	Reg.	Occ.	Ref.	Gan	Reg.	Occ.	Ref.	Gan	Reg.	Occ.	Ref.	Gan	Reg.	Occ.	Ref.	Gan	Reg.	Occ.	Ref.	Gan	Reg.	Occ.	Ref.	Gan	Reg.	Occ.	Ref.	Gan	Reg.	Occ.	Ref.
*10*	Acc.	0.90	**0.86**	0.84	0.43	0.11	**0.50**	0.45	0.36	0.03	0.64	0.58	**0.64**	0.00	**0.93**	0.79	0.71	0.92	**0.79**	**0.79**	0.50	0.66	0.93	**0.87**	0.71	0.74	**0.93**	0.68	0.79	0.21	**0.64**	0.66	**0.71**	0.24
	F_1_	0.86	**0.86**	0.75	0.60	0.15	**0.67**	0.57	0.32	0.05	0.69	0.55	**0.78**	0.00	**0.90**	0.86	0.83	0.84	**0.85**	0.81	0.67	0.72	0.93	**0.82**	0.74	0.78	**0.93**	0.70	0.88	0.25	0.75	0.69	**0.77**	0.36
*11*	Acc.	0.97	**1.00**	0.97	**1.00**	0.97	0.92	**0.97**	0.85	0.97	1.00	0.97	**1.00**	0.00	0.92	**0.97**	0.92	0.97	0.92	0.95	**1.00**	0.97	0.92	**0.97**	0.85	0.97	0.92	0.97	**1.00**	**1.00**	**1.00**	0.97	**1.00**	0.97
	F_1_	0.99	**1.00**	0.99	0.96	0.90	0.96	**0.97**	0.88	0.99	1.00	0.95	**1.00**	0.00	0.96	0.91	0.92	**0.99**	0.96	0.97	**1.00**	0.99	0.96	**0.99**	0.88	0.99	0.96	**0.97**	0.96	0.70	**1.00**	0.99	**1.00**	0.92
*14*	Acc.	1.00	**1.00**	0.88	1.00	**1.00**	**1.00**	**1.00**	0.29	1.00	0.57	**1.00**	1.00	0.81	**1.00**	**1.00**	**1.00**	0.94	**1.00**	**1.00**	0.86	1.00	**1.00**	1.00	0.57	1.00	**1.00**	**1.00**	**1.00**	0.94	0.86	**1.00**	0.29	1.00
	F_1_	1.00	0.78	0.93	0.58	**0.97**	0.78	**1.00**	0.27	1.00	0.73	**0.97**	0.67	0.90	0.93	**0.97**	0.64	0.94	**1.00**	**1.00**	0.71	0.91	**1.00**	1.00	0.62	0.91	0.78	**1.00**	0.78	0.91	0.80	**0.97**	0.33	0.86
*16*	Acc.	0.88	0.86	**0.94**	0.14	0.88	**1.00**	0.94	0.14	0.88	0.57	**0.88**	0.43	0.69	**1.00**	**1.00**	0.29	0.88	**1.00**	**1.00**	0.57	0.88	**1.00**	0.94	0.57	0.88	0.57	**0.94**	0.57	0.81	**1.00**	**1.00**	0.43	0.88
	F_1_	0.93	0.86	**0.91**	0.25	0.90	0.93	**0.94**	0.25	0.93	0.67	**0.90**	0.50	0.76	0.93	**0.97**	0.44	0.93	0.93	**0.97**	0.73	0.90	0.93	**0.97**	0.67	0.90	0.67	**0.97**	0.62	0.79	0.93	**1.00**	0.50	0.85
*20*	Acc.	0.82	0.00	0.18	**0.91**	0.03	**0.64**	0.61	0.73	0.39	0.00	0.00	0.73	0.00	0.73	0.85	**1.00**	0.79	0.82	0.82	0.73	**0.88**	0.73	0.79	0.55	**0.88**	0.09	0.30	**0.91**	0.00	0.55	0.39	0.64	**0.97**
	F_1_	0.89	0.00	0.31	**0.57**	0.06	**0.74**	0.71	0.47	0.19	0.00	0.00	0.59	0.00	0.84	**0.90**	0.61	0.87	**0.90**	0.86	0.67	0.62	0.84	0.87	0.55	**0.90**	0.17	0.45	**0.71**	0.00	**0.67**	0.57	0.45	0.27
*23*	Acc.	0.95	**1.00**	0.93	0.24	0.98	0.94	**0.95**	0.35	0.83	1.00	**0.98**	0.59	0.05	**1.00**	0.95	0.18	0.93	**1.00**	0.95	0.82	0.02	**1.00**	0.93	0.94	0.05	**1.00**	0.91	0.59	**1.00**	**1.00**	0.98	0.53	0.05
	F_1_	0.90	**0.81**	0.74	0.38	0.70	**0.91**	0.83	0.44	0.40	0.67	**0.56**	0.69	0.05	0.92	0.92	0.30	**0.93**	**0.94**	0.91	0.85	0.05	**0.92**	0.91	0.84	0.09	**0.79**	0.75	0.71	0.62	**0.92**	0.80	0.56	0.07
*30*	Acc.	0.84	**0.80**	0.60	0.86	0.70	**0.93**	0.87	0.21	0.24	0.60	0.16	**0.93**	0.00	0.87	**0.97**	0.86	0.76	0.87	0.87	**0.93**	0.89	0.87	**0.89**	0.79	0.84	0.87	0.81	**0.93**	0.30	**0.87**	0.76	0.86	0.70
	F_1_	0.84	**0.83**	0.68	0.71	0.48	**0.76**	0.67	0.32	0.36	0.64	0.22	**0.79**	0.00	**0.87**	0.77	0.80	0.78	0.81	**0.78**	0.74	0.75	0.87	**0.88**	0.73	0.75	**0.87**	0.73	0.84	0.27	**0.79**	0.68	0.77	0.68
*33*	Acc.	0.98	**1.00**	0.96	**1.00**	0.74	**1.00**	0.94	0.95	0.06	1.00	0.91	**1.00**	0.00	**1.00**	0.85	0.95	0.98	**1.00**	0.94	1.00	0.98	**1.00**	0.98	**1.00**	0.98	**1.00**	0.94	0.95	0.11	**1.00**	0.96	**1.00**	0.00
	F_1_	0.98	0.97	0.96	**1.00**	0.84	**0.97**	0.96	0.92	0.11	0.97	0.94	**1.00**	0.00	0.97	0.91	0.95	**0.98**	0.97	0.95	**1.00**	0.98	0.97	**0.98**	0.97	0.98	0.95	0.96	**0.97**	0.20	0.97	0.97	**1.00**	0.00
*34*	Acc.	0.96	0.90	0.89	0.95	**0.96**	0.90	**0.96**	0.95	0.19	0.90	0.46	**1.00**	0.00	0.90	**1.00**	0.95	0.94	0.95	0.94	**1.00**	0.90	0.95	0.89	**1.00**	0.85	0.90	0.98	**1.00**	0.54	0.90	0.96	**1.00**	0.00
	F_1_	0.96	0.94	0.91	**0.97**	0.96	0.94	0.94	**0.97**	0.32	0.94	0.89	**1.00**	0.00	0.94	0.93	**0.97**	0.95	**0.97**	0.94	**1.00**	0.93	0.97	0.92	**1.00**	0.91	0.94	0.96	**1.00**	0.70	0.94	0.95	**1.00**	0.00
*46*	Acc.	0.87	**1.00**	0.84	**1.00**	0.89	**1.00**	0.87	**1.00**	0.49	1.00	0.84	**1.00**	0.65	**1.00**	0.87	**1.00**	0.84	**1.00**	0.87	**1.00**	0.87	**1.00**	0.87	**1.00**	0.87	**1.00**	0.84	**1.00**	0.87	**1.00**	0.87	**1.00**	0.65
	F_1_	0.91	0.90	0.89	**0.97**	0.69	**0.93**	0.88	0.79	0.38	0.85	0.90	**1.00**	0.14	**0.97**	0.91	0.94	0.90	0.93	0.90	**0.97**	0.93	0.93	0.91	**0.97**	0.91	0.93	0.87	**0.97**	0.48	0.93	0.89	**1.00**	0.56
*48*	Acc.	0.92	0.87	**0.92**	0.80	0.28	**0.80**	0.69	0.73	0.13	0.80	0.56	**0.67**	0.00	**0.87**	0.59	0.80	0.85	**0.87**	0.85	**0.87**	0.85	0.87	**0.90**	0.80	0.90	0.87	**0.90**	0.73	0.05	**0.93**	0.87	0.73	0.13
	F_1_	0.88	**0.93**	0.78	0.89	0.43	**0.89**	0.76	0.76	0.22	0.89	0.58	**0.80**	0.00	**0.93**	0.74	0.86	0.87	**0.93**	0.87	**0.93**	0.72	**0.93**	**0.80**	0.89	0.74	**0.93**	0.86	0.85	0.10	**0.97**	0.88	0.85	0.23
*all*	WA	0.92	**0.86**	0.82	0.78	0.68	**0.87**	0.84	0.65	0.40	0.78	0.70	**0.83**	0.21	**0.93**	0.89	0.80	0.90	**0.93**	0.90	0.87	0.80	**0.93**	0.91	0.84	0.80	0.86	0.84	**0.87**	0.48	**0.89**	0.86	0.80	0.41

**Table A2:** Results on NTU-RGB+D dataset. “Bas.”/“Gan”/“Reg.”/“Occ.”/“Ref.” denote baseline/Generative adversarial networks/ Regression/training with occluded samples/reference case, Acc, F_1_ and WA denote Accuracy, F_1_ score and Weighted Accuracy, respectively. “None” denotes the case without occlusion. LA, RA, LL, RL denote the occlusion of left arm, right arm, left leg, right leg, respectively. Numbers in bold indicate best performance between “Rec.”/“Occ.”/“Ref.”. Numbers in italics denote the corresponding classes, as follows: 41: sneeze/cough, 42: staggering, 43: falling down, 44: headache, 45: chest pain, 46: back pain, 47: neck pain, 48: nausea/vomiting, 49: fan self, 103: yawn, 104: stretch oneself, 105: blow nose.

		None	LA				RA				LA+RA				LL				RL				LL+RL				LA+LL				RA+RL			
Class	Metric	Bas.	Gans	Reg.	Occ.	Ref.	Gans	Reg.	Occ.	Ref.	Gans	Reg.	Occ.	Ref.	Gans	Reg.	Occ.	Ref.	Gans	Reg.	Occ.	Ref.	Gans	Reg.	Occ.	Ref.	Gans	Reg.	Occ.	Ref.	Gans	Reg.	Occ.	Ref.
*41*	Acc.	0.89	0.74	0.70	**0.87**	0.73	0.32	0.51	**0.74**	0.14	0.16	0.57	**0.84**	0.11	0.74	0.73	**0.84**	0.24	**0.87**	0.65	0.81	0.62	**0.90**	0.87	0.74	0.24	0.77	0.70	**0.84**	0.16	0.16	**0.73**	0.71	0.11
	F_1_	0.82	0.55	0.66	0.73	**0.74**	0.31	0.54	**0.65**	0.19	0.17	0.58	**0.79**	0.11	**0.72**	0.70	0.71	0.35	**0.86**	0.66	0.74	0.59	**0.86**	0.78	0.73	0.38	0.55	0.68	**0.80**	0.24	0.26	**0.73**	0.73	0.19
*42*	Acc.	0.35	0.28	0.22	**0.47**	0.13	**0.38**	0.17	0.34	0.26	**0.56**	0.26	0.41	0.00	**0.44**	0.22	0.41	0.09	0.34	0.26	**0.44**	0.09	**0.31**	0.30	0.44	0.00	0.28	0.13	**0.41**	0.00	**0.56**	0.22	0.47	0.09
	F_1_	0.40	0.42	0.29	**0.42**	0.17	**0.46**	0.20	0.33	0.31	**0.48**	0.32	0.39	0.00	**0.53**	0.28	0.40	0.14	**0.46**	0.32	0.40	0.15	**0.41**	0.36	0.41	0.00	**0.39**	0.19	**0.39**	0.00	**0.55**	0.28	0.45	0.14
*43*	Acc.	0.66	**0.78**	0.62	0.59	0.72	**0.94**	0.66	0.53	0.83	0.50	**0.76**	0.69	0.62	**0.78**	0.69	0.47	0.52	**0.78**	0.76	0.69	0.76	**0.78**	0.76	0.69	0.72	0.69	**0.72**	0.69	0.62	0.69	**0.79**	0.69	0.55
	F_1_	0.61	**0.56**	0.46	0.50	0.46	0.41	**0.47**	0.42	0.35	0.24	**0.49**	0.51	0.29	**0.62**	0.53	0.41	0.38	**0.68**	0.54	0.46	0.27	**0.71**	0.57	0.50	0.36	**0.55**	0.51	0.59	0.31	0.41	**0.56**	0.51	0.26
*44*	Acc.	0.93	**0.90**	0.87	0.87	0.77	0.87	**0.90**	0.87	0.07	0.71	**0.90**	0.87	0.07	0.84	**0.90**	0.87	0.93	0.84	0.90	0.90	**0.97**	0.94	0.90	0.84	**1.00**	0.94	0.90	0.87	**0.97**	0.74	**0.90**	0.84	0.37
	F_1_	0.95	**0.95**	0.90	0.89	0.87	**0.93**	**0.93**	0.89	0.12	0.83	**0.95**	0.89	0.12	0.90	**0.93**	0.87	0.93	0.90	0.95	0.90	**0.95**	**0.95**	0.93	0.88	0.85	**0.97**	0.93	0.87	0.89	0.85	**0.93**	0.88	0.54
*45*	Acc.	0.41	**0.52**	0.18	0.28	0.46	0.03	0.23	0.19	**0.91**	0.32	0.23	0.28	**0.41**	**0.45**	0.23	0.25	0.14	**0.61**	0.23	0.34	0.59	**0.52**	0.50	0.22	0.50	**0.45**	0.18	0.41	0.14	0.23	0.41	0.16	**0.77**
	F_1_	0.51	**0.62**	0.22	0.27	0.40	0.06	0.24	0.23	**0.26**	0.05	0.23	0.25	**0.23**	**0.57**	0.26	0.25	0.17	**0.68**	0.26	0.29	0.32	**0.63**	0.47	0.26	0.23	**0.57**	0.21	0.38	0.19	0.31	0.40	0.19	**0.23**
*46*	Acc.	0.38	0.44	0.32	**0.47**	0.24	0.16	0.27	**0.47**	0.12	0.00	0.35	**0.56**	0.27	**0.56**	0.50	0.53	0.32	**0.56**	0.29	0.44	0.15	**0.50**	0.29	0.50	0.09	**0.53**	0.35	0.59	0.12	0.44	0.35	**0.66**	0.03
	F_1_	0.43	**0.49**	0.39	0.35	0.31	0.23	0.30	**0.33**	0.17	0.00	0.39	**0.41**	0.31	**0.59**	0.47	0.36	0.37	**0.57**	0.33	0.38	0.21	**0.50**	0.35	0.36	0.14	**0.55**	0.37	0.41	0.20	0.42	0.40	**0.42**	0.05
*47*	Acc.	0.53	**0.65**	0.40	0.42	0.63	**0.71**	0.27	0.42	0.27	**0.77**	0.27	0.58	0.07	**0.58**	0.33	0.45	0.33	**0.65**	0.27	0.52	0.37	**0.65**	0.33	0.65	0.13	0.58	0.40	**0.71**	0.33	**0.81**	0.33	0.65	0.43
	F_1_	0.64	**0.74**	0.53	0.55	0.73	**0.79**	0.39	0.55	0.28	**0.69**	0.38	0.65	0.11	**0.69**	0.45	0.56	0.43	**0.73**	0.39	0.58	0.48	**0.77**	0.45	0.63	0.21	**0.72**	0.51	0.70	0.43	**0.72**	0.45	0.63	0.36
*48*	Acc.	0.79	0.41	0.38	0.10	**0.42**	0.28	**0.38**	0.07	0.17	**0.62**	0.38	0.03	0.50	0.50	0.54	0.07	**0.67**	**0.66**	0.29	0.10	0.21	**0.50**	0.50	0.10	0.21	0.47	0.38	0.07	**0.63**	0.22	**0.38**	0.03	0.21
	F_1_	0.55	0.43	0.31	0.15	**0.47**	0.30	**0.32**	0.10	0.18	0.11	**0.31**	0.06	0.14	0.52	**0.39**	0.11	0.31	**0.58**	0.24	0.16	0.22	**0.46**	0.44	0.17	0.14	**0.54**	0.32	0.12	0.32	0.29	**0.32**	0.06	0.15
*49*	Acc.	0.68	**0.53**	0.48	0.44	0.48	0.25	**0.46**	0.38	0.05	0.19	0.36	**0.50**	0.00	**0.78**	0.46	0.28	0.77	**0.69**	0.48	0.56	0.16	**0.69**	0.48	0.44	0.41	0.53	0.46	0.38	**0.59**	0.25	**0.52**	0.41	0.23
	F_1_	0.59	**0.51**	0.42	0.44	0.47	0.34	**0.41**	0.39	0.07	0.23	0.36	**0.42**	0.00	**0.60**	0.44	0.35	0.46	**0.61**	0.42	0.47	0.24	**0.59**	0.45	0.38	0.31	**0.49**	0.43	0.40	0.40	0.25	**0.46**	0.37	0.26
*103*	Acc.	0.79	**0.81**	0.76	0.72	0.79	0.56	**0.82**	0.78	0.40	0.63	**0.76**	0.53	0.16	0.72	**0.76**	0.75	0.74	0.75	**0.84**	0.50	0.74	**0.84**	0.82	0.69	0.61	**0.81**	0.79	0.72	0.74	0.59	**0.79**	0.72	0.32
	F_1_	0.85	**0.87**	0.79	0.81	0.79	0.68	**0.84**	0.83	0.54	0.68	**0.79**	0.68	0.26	0.79	0.82	0.81	**0.84**	0.83	0.84	0.67	**0.85**	**0.89**	0.83	0.77	0.72	**0.87**	0.85	0.79	0.82	0.68	**0.83**	0.81	0.48
*104*	Acc.	0.97	0.84	**0.94**	0.75	0.74	0.78	**0.94**	0.75	0.21	0.00	**0.88**	0.69	0.00	**0.97**	0.88	0.84	0.21	**0.94**	**0.94**	0.63	0.15	**0.94**	0.88	0.78	0.03	0.91	**0.94**	0.78	0.12	0.56	**0.91**	0.81	0.32
	F_1_	0.96	0.90	**0.93**	0.81	0.82	0.85	**0.90**	0.76	0.34	0.00	**0.88**	0.79	0.00	**0.97**	0.90	0.84	0.33	**0.95**	0.93	0.75	0.24	**0.95**	0.90	0.86	0.06	0.88	**0.91**	0.83	0.21	0.71	**0.89**	0.85	0.48
*105*	Acc.	0.60	**0.78**	0.66	0.34	0.77	**0.69**	0.51	0.38	0.11	0.59	**0.63**	0.22	0.00	**0.81**	0.57	0.47	0.14	**0.84**	0.60	0.16	0.11	**0.84**	0.60	0.25	0.00	**0.75**	0.69	0.28	0.20	**0.66**	0.54	0.28	0.03
	F_1_	0.66	**0.67**	0.64	0.37	0.65	0.52	**0.54**	0.38	0.17	0.34	**0.65**	0.27	0.00	**0.70**	0.61	0.46	0.24	**0.68**	0.62	0.22	0.19	**0.67**	0.65	0.31	0.00	**0.65**	0.69	0.32	0.26	0.46	**0.60**	0.35	0.05
*all*	WA	0.68	**0.64**	0.57	0.53	0.59	0.50	**0.53**	0.49	0.27	0.35	**0.55**	0.52	0.16	**0.68**	0.59	0.52	0.44	**0.71**	0.57	0.51	0.41	**0.70**	0.62	0.53	0.33	**0.64**	0.58	0.56	0.40	0.49	**0.59**	0.53	0.25

**Table A3:** Results on UTKinect-Action3D dataset.“Bas.”/“Gan”/“Reg.”/“Occ.”/“Ref.” denote baseline/Generative adversarial networks/ Regression/training with occluded samples/reference case, Acc, F_1_ and WA denote Accuracy, F_1_ score and Weighted Accuracy, respectively. “None” denotes the case without occlusion. LA, RA, LL, RL denote the occlusion of left arm, right arm, left leg, right leg, respectively. Numbers in bold indicate best performance between “Rec.”/“Occ.”/“Ref.”. Numbers in italics denote the corresponding classes, as follows: 0: walk, 1: sitDown, 2: standUp, 3: pickUp, 4: carry, 5: throw, 6: push, 7: pull, 8: wavehands, 9: clapHands.

		None	LA				RA				LA+RA				LL				RL				LL+RL				LA+LL				RA+RL			
Class	Metric	Baseline	Gan	Reg.	Occ.	Ref.	Gan	Reg.	Occ.	Ref.	Gan	Reg.	Occ.	Ref.	Gan	Reg.	Occ.	Ref.	Gan	Reg.	Occ.	Ref.	Gan	Reg.	Occ.	Ref.	Gan	Reg.	Occ.	Ref.	Gan	Reg.	Occ.	Ref.
*0*	Acc.	0.50	**0.50**	0.40	0.30	0.30	**1.00**	0.40	0.30	0.00	**0.50**	0.40	0.30	0.00	**1.00**	0.40	0.30	0.20	**1.00**	0.40	0.40	0.50	**1.00**	0.40	0.30	0.20	0.50	0.40	0.50	**0.70**	**1.00**	0.40	0.40	0.00
	F_1_	0.48	**0.50**	0.43	0.35	0.31	**0.80**	0.43	0.35	0.00	**0.67**	0.39	0.26	0.00	**1.00**	0.43	0.31	0.09	**0.80**	0.43	0.41	0.41	**1.00**	0.43	0.28	0.07	**0.67**	0.43	0.44	0.33	**1.00**	0.39	0.43	0.00
*1*	Acc.	0.70	0.00	0.30	0.20	**0.60**	**0.50**	0.10	0.20	0.30	0.00	0.30	0.20	**0.90**	**1.00**	0.70	0.60	0.00	**0.50**	**0.50**	0.30	0.40	0.50	**0.60**	0.10	0.00	0.00	**0.40**	0.50	0.20	0.00	**0.30**	0.30	0.20
	F_1_	0.53	0.00	0.23	0.16	**0.15**	**0.50**	0.08	0.10	0.17	0.00	0.13	0.11	**0.18**	**0.80**	0.57	0.51	0.00	0.50	**0.39**	0.26	0.35	0.50	**0.43**	0.10	0.00	0.00	**0.32**	0.39	0.06	0.00	**0.21**	0.23	0.13
*2*	Acc.	0.80	**1.00**	0.80	0.70	0.00	**1.00**	0.80	0.50	0.00	**1.00**	0.90	0.80	0.00	**1.00**	0.80	0.80	0.10	**1.00**	0.70	0.80	0.60	**1.00**	0.70	0.60	0.00	**1.00**	0.90	0.90	0.20	**1.00**	0.90	0.40	0.00
	F_1_	0.87	**1.00**	0.76	0.66	0.00	0.67	**0.80**	0.41	0.00	0.80	**0.83**	0.76	0.00	**0.80**	0.80	0.67	0.13	**0.80**	0.75	0.66	0.63	**0.80**	0.75	0.46	0.00	0.80	0.79	**0.85**	0.27	**1.00**	0.89	0.26	0.00
*3*	Acc.	1.00	**0.50**	0.30	0.30	0.00	0.50	**0.60**	0.10	0.20	0.00	0.00	**0.30**	0.00	**1.00**	0.70	0.40	0.40	0.00	0.70	0.60	**1.00**	**1.00**	0.80	0.30	0.70	0.00	0.20	**0.70**	0.00	0.00	0.20	**0.30**	**0.30**
	F_1_	0.92	**0.50**	0.22	0.26	0.00	0.40	**0.43**	0.05	0.05	0.00	0.00	**0.33**	0.00	**1.00**	0.58	0.41	0.40	0.00	0.63	0.57	**0.69**	0.67	**0.72**	0.18	0.61	0.00	0.11	**0.63**	0.00	0.00	**0.16**	0.29	0.07
*4*	Acc.	0.90	0.00	0.00	**0.20**	0.00	0.00	0.40	0.10	**0.60**	0.00	0.00	**0.50**	0.00	0.50	0.60	0.20	**0.90**	**1.00**	0.60	0.60	0.50	**1.00**	**0.60**	0.10	**0.80**	**1.00**	0.10	0.50	0.00	0.50	0.30	0.10	**0.60**
	F_1_	0.93	0.00	0.00	**0.11**	0.00	0.00	**0.40**	0.13	0.14	0.00	0.00	**0.41**	0.00	0.50	0.57	0.13	**0.73**	**0.57**	0.60	0.57	0.39	**1.00**	**0.57**	0.08	**0.59**	**0.50**	0.08	0.46	0.00	**0.40**	0.20	0.13	0.14
*5*	Acc.	1.00	**1.00**	0.80	0.50	0.00	0.00	**0.80**	0.50	0.00	0.00	**0.80**	0.40	0.00	0.00	0.80	0.50	**0.90**	0.50	**0.80**	0.40	0.70	0.50	**0.80**	0.50	**0.80**	0.00	**0.80**	0.50	0.10	**1.00**	0.80	0.50	0.00
	F_1_	0.92	**1.00**	0.69	0.49	0.00	0.00	**0.69**	0.43	0.00	0.00	**0.72**	0.47	0.00	0.00	**0.69**	0.50	0.58	0.67	**0.69**	0.43	0.65	0.67	**0.69**	0.40	0.59	0.00	**0.73**	0.60	0.13	**1.00**	0.76	0.43	0.00
*6*	Acc.	1.00	**1.00**	0.80	0.50	0.00	1.00	**0.80**	0.70	0.00	**1.00**	0.90	0.80	0.00	**1.00**	0.90	0.80	**1.00**	**1.00**	0.80	0.80	0.90	**1.00**	0.80	0.70	0.80	**1.00**	0.80	1.00	0.20	**1.00**	1.00	0.70	0.00
	F_1_	1.00	**1.00**	0.80	0.49	0.00	1.00	**0.80**	0.66	0.00	**0.80**	0.93	0.61	0.00	0.80	**0.93**	0.72	0.79	**1.00**	0.76	0.68	0.81	**1.00**	0.76	0.63	0.51	0.80	0.83	**0.86**	0.13	**1.00**	0.96	0.63	0.00
*7*	Acc.	0.40	0.00	0.50	**0.60**	**0.70**	0.00	**0.40**	0.30	0.00	**1.00**	0.20	0.60	0.00	0.00	**0.40**	0.30	0.00	0.00	**0.20**	0.70	0.10	0.00	0.30	**0.40**	0.00	0.00	**0.40**	0.30	**0.40**	**0.50**	0.40	**0.50**	0.00
	F_1_	0.43	0.00	**0.25**	0.22	0.23	0.00	**0.28**	0.21	0.00	**0.36**	0.17	0.35	0.00	0.00	**0.40**	0.19	0.00	0.00	**0.12**	0.54	0.10	0.00	0.27	**0.34**	0.00	0.00	**0.24**	0.21	0.11	**0.67**	0.34	0.28	0.00
*8*	Acc.	0.60	**1.00**	0.40	0.50	0.00	1.00	**0.40**	0.30	0.00	**1.00**	0.20	0.50	0.00	**1.00**	0.40	0.60	0.50	**1.00**	0.40	0.60	0.40	**1.00**	0.40	0.50	0.30	**1.00**	0.30	0.50	0.20	**1.00**	0.40	0.40	0.00
	F_1_	0.52	**0.80**	0.36	0.44	0.00	1.00	**0.36**	0.29	0.00	**1.00**	**0.16**	0.47	0.00	**1.00**	0.27	0.55	0.40	**1.00**	0.36	0.55	0.43	**1.00**	0.29	0.53	0.27	**0.80**	0.26	0.53	0.27	**1.00**	0.36	0.44	0.00
*9*	Acc.	1.00	**1.00**	0.70	0.40	0.00	1.00	**1.00**	0.30	0.40	**1.00**	0.30	1.00	0.00	**1.00**	**1.00**	0.70	**1.00**	**1.00**	**1.00**	**1.00**	**1.00**	**1.00**	1.00	0.90	**1.00**	**1.00**	0.80	**1.00**	0.20	**1.00**	1.00	0.80	0.00
	F_1_	0.87	**0.57**	0.52	0.23	0.00	1.00	**0.83**	0.10	0.47	**1.00**	0.13	0.88	0.00	**1.00**	0.87	0.59	0.69	**1.00**	0.87	**0.96**	0.96	**1.00**	0.87	0.81	0.74	**1.00**	0.58	**0.92**	0.07	**1.00**	0.87	0.76	0.00
*all*	WA	0.79	**0.60**	0.50	0.42	0.16	0.60	**0.57**	0.33	0.15	**0.55**	0.40	0.54	0.09	**0.75**	0.67	0.52	0.50	**0.70**	0.61	0.62	0.61	**0.80**	0.64	0.44	0.46	0.55	0.51	**0.64**	0.22	**0.70**	0.57	0.44	0.11

**Table A4:** Results on SYSU-3D-HOI dataset. “Bas.”/“Gan”/“Reg.”/“Occ.”/“Ref.” denote baseline/Generative adversarial networks/ Regression/training with occluded samples/reference case, Acc, F_1_ and WA denote Accuracy, F_1_ score and Weighted Accuracy, respectively. “None” denotes the case without occlusion. LA, RA, LL, RL denote the occlusion of left arm, right arm, left leg, right leg, respectively. Numbers in bold indicate best performance between “Rec.”/“Occ.”/“Ref.”. Numbers in italics denote the corresponding classes, as follows: 1: drinking, 2: pouring, 3: calling phone, 4: playing phone, 5: wearing backpacks, 6: packing backpacks, 7: sitting chair, 8: moving chair, 9: taking out wallet, 10: taking from wallet, 11: mopping, 12: sweeping.

		None	LA				RA				LA+RA				LL				RL				LL+RL				LA+LL				RA+RL			
Class	Metric	Baseline	Gan	Reg.	Occ.	Ref.	Gan	Reg.	Occ.	Ref.	Gan	Reg.	Occ.	Ref.	Gan	Reg.	Occ.	Ref.	Gan	Reg.	Occ.	Ref.	Gan	Reg.	Occ.	Ref.	Gan	Reg.	Occ.	Ref.	Gan	Reg.	Occ.	Ref.
*1*	Acc.	0.10	0.00	0.00	**0.13**	0.00	0.00	0.05	**0.13**	0.00	**0.25**	0.00	**0.25**	0.00	0.00	0.00	**0.10**	0.00	0.00	0.10	**0.28**	0.00	0.00	0.05	**0.13**	0.00	**0.00**	0.00	**0.28**	0.00	0.00	0.00	**0.05**	0.00
	F_1_	0.10	0.00	0.00	**0.14**	0.00	0.00	0.06	**0.07**	0.00	0.22	0.00	**0.25**	0.00	0.00	0.00	**0.11**	0.00	0.00	0.10	**0.28**	0.00	0.00	0.08	**0.11**	0.00	0.00	0.00	**0.28**	0.00	0.00	0.00	**0.06**	0.00
*2*	Acc.	0.65	0.25	**0.45**	0.43	0.00	0.25	**0.65**	0.13	0.05	0.25	0.00	**0.50**	0.00	**0.50**	0.45	0.40	0.45	0.25	**0.55**	0.53	0.20	0.50	**0.55**	0.28	0.10	**0.50**	**0.50**	0.48	0.30	0.25	0.00	**0.28**	0.00
	F_1_	0.59	0.22	0.36	**0.46**	0.00	0.33	**0.63**	0.12	0.08	0.40	0.00	**0.54**	0.00	0.40	0.44	**0.45**	0.34	0.33	0.56	**0.57**	0.23	**0.44**	**0.53**	0.34	0.04	0.33	**0.48**	0.49	0.16	0.40	0.00	**0.19**	0.00
*3*	Acc.	0.80	0.50	**0.75**	0.60	0.55	0.75	**0.85**	0.48	0.05	0.50	0.60	**0.68**	0.45	0.50	**0.70**	0.58	0.00	0.50	**0.85**	0.68	0.00	0.25	**0.80**	0.48	0.00	0.50	0.60	**0.65**	0.25	0.25	**0.55**	0.55	0.10
	F_1_	0.77	0.67	**0.69**	0.54	0.21	0.67	**0.77**	0.44	0.05	**0.67**	0.57	0.59	0.12	0.57	**0.65**	0.56	0.00	0.67	**0.71**	0.59	0.00	0.33	**0.73**	0.46	0.00	**0.57**	0.56	0.59	0.18	0.33	**0.61**	0.50	0.06
*4*	Acc.	0.75	**0.75**	0.70	0.58	0.05	0.75	**0.80**	0.53	0.30	**0.75**	**0.75**	0.65	0.00	**0.75**	**0.75**	0.68	0.40	**0.75**	**0.75**	0.65	0.25	**0.75**	0.70	0.58	0.20	**0.75**	**0.75**	0.70	0.25	0.75	**0.80**	0.68	0.25
	F_1_	0.80	**0.75**	0.73	0.61	0.08	**0.86**	0.84	0.57	0.43	**0.86**	0.76	0.71	0.00	**0.86**	0.79	0.70	0.52	**0.86**	0.78	0.72	0.34	**0.86**	0.75	0.62	0.29	**0.75**	**0.75**	0.76	0.27	**0.86**	0.76	0.66	0.31
*5*	Acc.	0.55	**0.50**	0.45	0.28	0.10	**1.00**	0.40	0.53	0.40	**0.75**	0.50	0.40	0.00	**0.75**	0.45	0.38	0.40	**1.00**	0.40	0.40	0.00	**0.75**	0.50	0.25	0.20	**0.75**	0.30	0.33	0.50	**1.00**	0.50	0.30	0.10
	F_1_	0.48	**0.50**	0.34	0.21	0.10	**0.80**	0.35	0.40	0.15	**0.67**	0.30	0.38	0.00	**0.67**	0.38	0.32	0.10	**0.80**	0.39	0.41	0.00	**0.67**	0.39	0.23	0.19	**0.86**	0.28	0.32	0.20	**0.67**	0.44	0.31	0.07
*6*	Acc.	0.40	**0.75**	0.25	0.28	0.05	**0.50**	0.25	0.05	0.00	0.00	0.00	**0.30**	0.00	**0.50**	0.30	0.33	0.00	**0.75**	0.35	0.28	0.00	**0.75**	0.25	0.25	0.00	**0.50**	0.30	0.33	0.00	**0.50**	0.25	0.43	0.00
	F_1_	0.38	**0.60**	0.31	0.27	0.08	**0.50**	0.28	0.03	0.00	0.00	0.00	**0.28**	0.00	**0.40**	0.27	0.31	0.00	**0.60**	0.32	0.26	0.00	**0.60**	0.18	0.22	0.00	**0.40**	0.27	0.30	0.00	**0.40**	0.20	0.38	0.00
*7*	Acc.	0.45	0.25	0.40	0.43	**0.50**	0.25	**0.35**	0.33	0.30	0.00	0.15	**0.65**	0.00	0.25	0.40	**0.43**	0.00	0.25	**0.45**	0.68	0.00	0.25	**0.45**	0.45	0.00	0.25	0.35	**0.55**	0.20	0.25	0.35	**0.53**	0.00
	F_1_	0.54	**0.40**	0.35	0.39	0.18	**0.40**	0.36	0.29	0.19	0.00	0.20	**0.61**	0.00	0.40	**0.47**	0.42	0.00	0.29	**0.46**	0.60	0.00	0.29	**0.42**	0.42	0.00	0.40	0.41	**0.52**	0.06	0.33	0.40	**0.43**	0.00
*8*	Acc.	0.90	**1.00**	0.85	0.80	0.10	**1.00**	0.85	0.75	0.65	**1.00**	0.75	0.75	0.00	**1.00**	0.90	0.78	0.80	**1.00**	0.95	0.80	0.60	**1.00**	0.95	0.73	0.60	**1.00**	0.90	0.78	0.50	**1.00**	0.85	0.80	0.35
	F_1_	0.88	**1.00**	0.81	0.79	0.13	**1.00**	0.83	0.74	0.71	**1.00**	0.77	0.77	0.00	**1.00**	0.84	0.79	0.75	**0.89**	0.87	0.79	0.58	**1.00**	0.88	0.74	0.63	**1.00**	0.88	0.81	0.56	**1.00**	0.80	0.84	0.46
*9*	Acc.	0.65	**1.00**	0.30	0.35	0.50	**0.75**	0.25	0.30	0.40	**1.00**	0.20	0.33	0.80	**1.00**	0.40	0.40	0.00	**1.00**	0.35	0.38	0.00	**1.00**	0.35	0.28	0.00	**1.00**	0.40	0.35	0.10	**1.00**	0.25	0.30	0.65
	F_1_	0.67	**0.62**	0.39	0.31	0.19	**0.60**	0.31	0.28	0.31	**0.73**	0.28	0.34	0.19	**0.73**	0.47	0.30	0.00	**0.80**	0.45	0.39	0.00	**0.67**	0.38	0.24	0.00	**0.80**	0.44	0.31	0.12	**0.67**	0.26	0.25	0.12
*10*	Acc.	0.30	0.00	0.15	**0.33**	0.00	0.25	**0.30**	0.13	0.05	0.00	0.00	0.28	0.00	0.00	0.30	**0.40**	0.00	0.25	**0.30**	0.28	0.00	0.25	0.25	**0.33**	0.00	0.25	**0.30**	0.30	0.05	**0.50**	0.25	0.15	0.05
	F_1_	0.24	0.00	0.12	**0.27**	0.00	**0.22**	0.21	0.07	0.06	0.00	0.00	0.22	0.00	0.00	0.20	**0.29**	0.00	0.20	**0.22**	0.21	0.00	0.25	0.18	**0.26**	0.00	0.29	**0.31**	0.24	0.06	**0.36**	0.20	0.12	0.08
*11*	Acc.	0.45	**0.50**	0.40	0.30	0.00	**0.50**	0.45	0.25	0.10	**0.50**	0.30	0.35	0.00	0.50	**0.60**	0.20	0.00	**0.50**	**0.50**	0.33	0.15	0.50	**0.55**	0.33	0.00	**0.50**	0.25	0.28	0.00	0.25	**0.50**	0.23	0.00
	F_1_	0.34	**0.31**	0.31	0.24	0.00	0.31	**0.32**	0.14	0.10	**0.33**	0.23	0.26	0.00	0.36	**0.43**	0.15	0.00	0.40	**0.44**	0.25	0.10	0.36	**0.39**	0.31	0.00	**0.31**	0.17	0.21	0.00	0.18	**0.29**	0.19	0.00
*12*	Acc.	0.45	0.25	0.35	**0.40**	0.00	0.00	0.25	0.40	**0.35**	0.25	**0.55**	0.23	0.00	0.25	0.30	0.35	**0.40**	0.00	0.20	0.35	**0.90**	0.00	0.10	0.38	**0.80**	0.00	**0.50**	0.23	0.20	0.00	**0.35**	0.20	0.10
	F_1_	0.45	0.40	0.34	**0.41**	0.00	0.00	0.22	**0.45**	0.18	0.20	**0.24**	0.24	0.00	0.29	**0.25**	0.39	0.09	0.00	0.15	0.38	**0.18**	0.00	0.10	0.32	0.16	0.00	**0.38**	0.25	0.14	0.00	**0.23**	0.19	0.02
*all*	WA	0.54	**0.48**	0.42	0.41	0.15	**0.50**	0.45	0.33	0.22	0.44	0.32	**0.45**	0.10	**0.50**	0.46	0.42	0.20	**0.52**	0.48	0.47	0.18	**0.50**	0.46	0.37	0.16	**0.50**	0.43	0.44	0.20	**0.48**	0.39	0.37	0.13
